# Supplementary material for: Re-engagement in care of people living with HIV lost to follow-up after initiation of antiretroviral therapy in Mali: Who returns to care?
Source: PLoS One. 2020 Sep 10;15(9):e0238687. doi: 10.1371/journal.pone.0238687 (PMC7482938; doi:10.1371/journal.pone.0238687)
Supplement: S1 Table — (DOCX) [file pone.0238687.s001.docx]

**S1 Table.** Characteristics at the start of antiretroviral therapy (ART), duration of ART until LTFU and the estimated 12-month change in CD4 count between ART initiation and LTFU of individuals lost to follow-up (LTFU) before 12/31/2013, overall and by group (“Extended LTFU” and “Return to care”) (data not imputed).

| Characteristics | LTFU before 12/31/2013  N = 3,650 | Extended LTFU  N = 1,975 | Return to care  N = 1,675 |
| --- | --- | --- | --- |
|  | *n (%)* | *n (%)* | *n (%)* |
| Sex and pregnancy  Men  Non-pregnant women  Pregnant women | 1,270 (34.8)  2,097 (57.5)  283 (7.7) | 723 (36.6)  1,171 (59.3)  81 (4.1) | 547 (32.7)  926 (55.3)  202 (12.0) |
| Age (years)  < 30  [30-40[  ≥ 40 | 35 (29-43)  1,036 (28.4)  1,434 (39.3)  1,180 (32.3) | 35 (29-43)  551 (27.9)  761 (38.5)  663 (33.6) | 35 (28-42)  485 (29.0)  673 (40.2)  517 (30.8) |
| WHO stage and CD4 count (cells/µL) (missing WHO stage, 9%; missing CD4 at J0, 14%)  WHO stage 3-4 and CD4 < 200  WHO stage 3-4 and CD4 ≥ 200  WHO stage 1-2 and CD4 < 200  WHO stage 1-2 and CD4 ≥ 200  WHO stage 3-4 and CD4 missing  WHO stage 1-2 and CD4 missing  WHO stage missing and CD4 < 200  WHO stage missing and CD4 ≥ 200 | 781 (21.4)  376 (10.3)  888 (24.3)  745 (20.4)  255 (7.0)  251 (6.9)  236 (6.5)  118 (3.2) | 480 (24.3)  214 (10.8)  403 (20.4)  317 (16.0)  145 (7.3)  160 (8.1)  179 (9.1)  77 (4.0) | 301 (18.0)  162 (9.7)  485 (29.0)  428 (25.5)  110 (6.6)  91 (5.4)  57 (3.4)  41 (2.4) |
| Antiretroviral Therapy (ART)  AZT or d4T+3TC+NVP or EFV  TDF+3TC or FTC+NVP or EFV  Other^a^ | 2,958 (81.0)  429 (11.8)  263 (7.2) | 1,519 (76.9)  297 (15.0)  159 (8.1) | 1,439 (85.9)  132 (7.9)  104 (6.2) |
| Periods of ART initiation  2006-2007  2008-2009  2010-2012 | 1,415 (38.8)  870 (23.8)  1,365 (37.4) | 598 (30.3)  391 (19.8)  986 (49.9) | 817 (48.8)  479 (28.6)  379 (22.6) |
| Marital status  Monogamous^b^  Polygamous  Single  Widowed  Divorced  Missing | 1,568 (43.0)  711 (19.5)  477 (13.1)  461 (12.6)  194 (5.3)  239 (6.5) | 869 (44.0)  358 (18.1)  251 (12.7)  221 (11.2)  97 (4.9)  179 (9.1) | 699 (41.7)  353 (21.1)  226 (13.5)  240 (14.3)  97 (5.8)  60 (3.6) |
| Educational level  None  Primary/Koranic  Secondary  Higher  Missing | 1,460 (40.0)  907 (24.8)  473 (13.0)  174 (4.8)  636 (17.4) | 784 (39.7)  443 (22.4)  221 (11.2)  81 (4.1)  446 (22.6) | 676 (40.4)  464 (27.7)  252 (15.0)  93 (5.6)  190 (11.3) |

| Characteristics | LTFU before 12/31/2013  N = 3,650 | Extended LTFU  N = 1,975 | Return to care  N = 1,675 |
| --- | --- | --- | --- |
|  | *n (%)* | *n (%)* | *n (%)* |
| Professional activity^c^  Housewife  Public sector staff  Private sector staff  Self-employed  Farmer/Fisherman  Other  Missing | 1,435 (39.3)  209 (5.7)  691 (18.9)  521 (14.3)  243 (6.7)  433 (11.9)  118 (3.2) | 768 (38.9)  104 (5.3)  341 (17.3)  287 (14.5)  164 (8.3)  223 (11.3)  88 (4.4) | 667 (39.8)  105 (6.3)  350 (20.9)  234 (14.0)  79 (4.7)  210 (12.5)  30 (1.8) |
| Regions, care centre & distance (km)  Bamako outpatient clinics  Bamako hospitals & < 5  Bamako hospitals & [5-50[  Bamako hospitals & ≥ 50  Regional outpatient clinics & < 5  Regional outpatient clinics & [5-50[  Regional outpatient clinics & ≥ 50  Regional hospitals & [5-50[  Regional hospitals & ≥ 50 | 1,839 (50.4)  119 (3.2)  649 (17.8)  107 (2.9)  191 (5.2)  112 (3.1)  58 (1.6)  426 (11.7)  149 (4.1) | 834 (42.2)  51 (2.6)  349 (17.7)  52 (2.6)  95 (4.8)  74 (3.8)  37 (1.9)  355 (17.9)  128 (6.5) | 1,005 (60.0)  68 (4.0)  300 (17.9)  55 (3.3)  96 (5.7)  38 (2.3)  21 (1.3)  71 (4.2)  21 (1.3) |
| Duration of ART until LTFU (month)    < 6  [6-12[  ≥ 12 | 11 (5-22)  1,049 (28.7)  881 (24.2)  1,720 (47.1) | 10 (5-25)  711 (36.0)  384 (19.4)  880 (44.6) | 12 (7-21)  338 (20.2)  497 (29.7)  840 (50.1) |
| 12-month change in CD4 count (cell/µL) (missing CD4 at LTFU, 44%)  < 50  ≥ 50  Missing | 1,416 (38.8)  633 (17.3)  1,601 (43.9) | 887 (44.9)  148 (7.5)  940 (47.6) | 529 (31.6)  485 (29.0)  661 (39.4) |

Data are counts (proportions) and medians (interquartile range). Abbreviations: LTFU, loss to follow-up; EFV, efavirenz; NVP, nevirapin; AZT, zidovudine; d4T, stavudine; 3TC, lamivudine; TDF, tenofovir; FTC, emtricitabine; ABC, abacavir; ddI, didanosine; LPV, lopinavir; ATV; atazanavir; IDV, indinavir; SQV, saquinavir; r, ritonavir NRTI, nucleoside reverse transcriptase inhibitors; PI, protease inhibitors. ^a^Other: NVP or EFV plus 2 NRTI (ABC/3TC, ddI/3TC, TDF/ddI and TDF/ABC), PI (LPVr, ATVr, IDVr and SQVr) plus 2 NRTI (nucleoside reverse transcriptase inhibitors) or 3 NRTIs combination. ^b^Married or living with a partner. ^c^Professional activities: professional activity categories combined types and sectors of employment, housewife without any professional activity, public or private sector salaried employment, various non-salaried activities, such as being self-employed, farmer/fisherman, and other (unemployed including students).
