# Supplementary material for: Re-engagement in care of people living with HIV lost to follow-up after initiation of antiretroviral therapy in Mali: Who returns to care?
Source: PLoS One. 2020 Sep 10;15(9):e0238687. doi: 10.1371/journal.pone.0238687 (PMC7482938; doi:10.1371/journal.pone.0238687)
Supplement: S2 Table — (DOCX) [file pone.0238687.s002.docx]

**S2 Table.** Multivariable Hazards Ratio (HRs) for re-engagement in the 36 months after loss to follow-up (LTFU) in each expertise level of care centre according to the region (Cox model with imputed data).

| Expertise level of care centre* | Extended LTFU | Return to care | Multivariable | |
| --- | --- | --- | --- | --- |
|  |  |  | **HR (95% CI)** | ***P*** |
| Outpatient clinics  Regions  Bamako | **N=1,040** | **N=1,160** | 1  1.21(0.99-1.49) | *0.0675* |
|  | **n (%)** | **n (%)** |  |  |
|  | 206 (19.8)  834 (80.2) | 155 (13.4)  1,005 (86.6) |  |  |
| Hospitals  Regions  Bamako | **N=935** | **N=515** | 1  2.20 (1.71-2.84) | *< 0.0001* |
|  | **n (%)** | **n (%)** |  |  |
|  | 483 (51.7)  452 (48.3) | 92 (17.9)  423 (82.1) |  |  |

***** Adjusted on sex & pregnancy, age, WHO stage and CD4 count, period of ART initiation, marital status, education level, professional activity, distance from home to care centre and a combined variable of the duration of ART until LTFU (months) and the estimation of the 12-month change in CD4 count (cells/µL).
