# Supplementary material for: Re-engagement in care of people living with HIV lost to follow-up after initiation of antiretroviral therapy in Mali: Who returns to care?
Source: PLoS One. 2020 Sep 10;15(9):e0238687. doi: 10.1371/journal.pone.0238687 (PMC7482938; doi:10.1371/journal.pone.0238687)
Supplement: Attachment — Submitted filename: Review report Re-engagement.pdf [file pone.0238687.s003.pdf]

Review report

In this paper, the authors provide interesting data about re-engagement in care of PLHIV after a period of LTFU. However, the study has several limitations and the statistical analysis has to be improved to make the conclusion more reliable. Furthermore, Authors have published results on LTFU in 2019: and they described LTFU in their papers: The current paper might be concentrated in re-engagement as primary outcome instead of repeating data on LTFU in the same population:

https://pubmed.ncbi.nlm.nih.gov/30270487-risk-factors-for-loss-to-follow-up-transfer-or-death-among-people-living-with-hiv-on-their-first-antiretroviral-therapy-regimen-in-mali/?from_term=Risk+factors+for+loss+to+follow+up%2C+transfer+or+death+among+people+living+on+their+first+antiretroviral+therapy+regimen+in++Mali&from_pos=1

The manuscript should more clearly compare the re-engagement incidence estimates of PLHIV in care of this study with other African studies and studies from other continents.

The Authors should note that PLOS One publication criteria state that if a submitted study replicates or is very similar to previous work, authors must provide a sound scientific rationale for the submitted work and clearly reference and discuss the existing literature. Submissions that replicate or are derivative of existing work will likely be rejected if authors do not provide adequate justification:

(https://journals.plos.org/plosone/s/criteria-for-publication#loc-2)

Method:-How did they measure the socio-economic status? We did not found such data in the manuscript. They have reported Professional activity: Did they use that as socio-economic status?

Method: How education is categorized and defined needs to be added in the method section or as footnote. When Authors are reporting secondary: is this the attained or the achieved level?

To analyze the Re-engagement issue: How authors did measure the duration from LTFU and re-engagement: What was the end date since this is the outcome.

Method:-: The information on 12-month change in CD4 count, does not make much sense. Did they a strong evidence to use this way to calculate this variable?

Method:- Authors should report the analytic approaches they used in the method section: which statistics tests, what was the significance alpha value . Furthermore, in my opinion it would be important to indicate Which confounders were used in the adjusted models? Authors should define clearly the threshold for significance (alpha).

For step-wise multiple regression analyses: Authors should report the alpha level used; discuss whether the variables were assessed for collinearity and interaction; describe the variable selection process by which the final model was developed (e.g., forward-stepwise; ect…).

Since Authors compare median , they should , detail any post hoc tests that were performed.

Manuscripts submitted to *PLOS ONE* are expected to report statistical methods in sufficient detail for others to replicate the analysis performed. Ensure that results are rigorously reported in accordance with community standards and that the statistical methods employed are appropriate for the study design.

- Results, Table1 and 3 must be rewritten : It would be better to mention the exact p-values for each category instead of reporting overall p-value. for example: when the authors reported HR: 1.03(0.95-1.11) and used p-value of 0.0013 for 1-2 and CD4≥200: this will confuse reader. Tables 1 and 3 must be totally rewritten.

- Results, Why did the authors categorize age and did not use it as continuous variable? Furthermore, the authors should revise the categories of variables : Age and Regions, care and distance: There are overlapping between: 30-40 and ≥40. Same thing with distance

- Results: he authors have introduced sex and professional activity in their models: Did they check for multicollinearity? Did they check the VIF?

Results: The authors mention several information (data) that were not mentioned in the any tables or figures: They should provide Supporting information(*"S" and number. For example, "S1 Appendix" and "S2 Appendix," "S1 Table" and "S2 Table," and so forth*) to support the statements provided in this manuscript.

- Discussion: The discussion needs to be rewritten. The authors mention several

information (data) that were not mentioned in the results and the comparison to other studies is insufficient.

- Conclusion in the abstract is reporting ART adherence and transportation issues which were not collected and analyzed in this study. Conclusions must be presented in an appropriate fashion and must be supported by the data

- References must be rewritten according to PLOS One policies: PLOS uses the numbered citation (citation-sequence) method and first six authors, et al.
- Authors are citing unpublished work in the manuscript (LTFU rates did not differ according to the distance between home and the care centre among the PLHIV followed at Bamako outpatient clinics(data not shown)):
- According to the Plos One policies: Do not cite the following sources in the reference list: Unavailable and unpublished work, including manuscripts that have been submitted but not yet accepted (e.g., "unpublished work," "data not shown"). Instead, include those data as supplementary material or deposit the data in a publicly available database.

Conclusion: MAJOR REVISION
