# Supplementary material for: Re-engagement in care of people living with HIV lost to follow-up after initiation of antiretroviral therapy in Mali: Who returns to care?
Source: PLoS One. 2020 Sep 10;15(9):e0238687. doi: 10.1371/journal.pone.0238687 (PMC7482938; doi:10.1371/journal.pone.0238687)
Supplement: Attachment — Submitted filename: Review report Re-engagement Paper.docx [file pone.0238687.s005.docx]

**Review report**

The Authors did not address adequately to our comments therefore, We are maintaining the decision of major revision. The Authors did not provide adequate justification. This submission is replicating or derive from existing work. The Plos One Policy is clear for this case and Editor has to appreciate.

Authors have published results on LTFU in 2019: and they described LTFU in their papers: The current paper might be concentrated in re-engagement as primary outcome instead of repeating data on LTFU in the same population:

<https://pubmed.ncbi.nlm.nih.gov/30270487-risk-factors-for-loss-to-follow-up-transfer-or-death-among-people-living-with-hiv-on-their-first-antiretroviral-therapy-regimen-in-mali/?from_term=Risk+factors+for+loss+to+follow+up%2C+transfer+or+death+among+people+living+on+their+first+antiretroviral+therapy+regimen+in++Mali&from_pos=1>

The Authors should note that PLOS One publication criteria state that if a submitted study replicates or is very similar to previous work, authors must provide a sound scientific rationale for the submitted work and clearly reference and discuss the existing literature. Submissions that replicate or are derivative of existing work will likely be rejected if authors do not provide adequate justification:

(<https://journals.plos.org/plosone/s/criteria-for-publication#loc-2>)

Method:-How did they measure the socio-economic status? We did not found such data in the manuscript. They have reported Professional activity: Did they use that as socio-economic status?

The authors said that , they removed all the socioeconomic terms in their manuscript. Socio-economic terms are important in terms of controlling confounding. They have to provide rationale and how they have define and collect such variables instead of to remove. This is reduce the quality of their analysis.

In the method section: Authors should explain why they define periods of ART initiation in such period. Did they do in term of national guidelines?

Authors did not address to this comment: «To analyze the Re-engagement issue: How authors did measure the duration from LTFU and re-engagement: What was the end date since this is the outcome.» Readers will need to know How they measure the duration from LTFU and re-engagement. If the definition is not clear this can introduce bias and misclassification. For the patients who were definitively LTFU during the subsequent three-year follow-up period, How did Authors calculate the duration for them. The start date and End point are not clear: This still some confusion. If this is not clear; Authors should use logistic regression instead of Cox regression?

Authors said: «We assessed factors associated with re-engagement in care in PLHIV lost to follow-up. For this analysis, PLHIV who were lost to follow-up after December 31, 2013 were excluded to allow sufficient time to re-engage in care before the last database update (03/31/2015). » and «This study included all HIV-1-infected adults, aged ≥ 18 years, who began ART between January 1, 2006 and December 31, 2012 at one of the 16 care centres contributing to the ESOPE database, thus beginning treatment at least two years before the last database update (31 March 2015), and who returned for their one-month visit.» This can make confusion

Authors said: «We fitted linear regression, logistic regression, and multinomial regression models for CD4 counts at the start of ART and at LTFU (fourth root), WHO stage (1-2 or 3-4), and educational level (none, primary/koranic, secondary and higher), respectively. » We did not found any results reported to this analysis.

Authors said that they did not look for confounding variables. If this is the case, What will be the validity of such study: Did authors assess the internal validity of their study? During this process, authors should analyze type of errors which could occur in their study:

- Random errors( sample size and sampling procedure)

- Selection Bias (LFU in the RCT)

- Information Bias: How the data was measured; (observer, instrument, observed and environment) . It can be : error measurement, improper diagnostic criteria, omissions, imprecise measurement. To assess it we use sensitivity and specificity to determine the degree of misclassification ( That why we need clear of how they measure the duration from LTFU and re-engagement)

- Confounding: we can control it in two stage:

in design: by randomization, stratification, matching (case-control) and restriction( by inclusion and exclusion criteria )

in Analysis: Stratification or by using multivariate technique

Authors assessed the interactions between several variables without providing the reason of doing that.

Confounding factors is what looks like a causal relationship between a supposed factor and an outcome( disease, other event,..) may be due to another factor not taken into consideration.

Manuscripts submitted to *PLOS ONE* are expected to report statistical methods in sufficient detail for others to replicate the analysis performed. Ensure that results are rigorously reported in accordance with community standards and that the statistical methods employed are appropriate for the study design.

I would request that Authors put publically their dataset for next version.

Authors said : «The study was approved by the national AIDS programme in Mali (approval letter available). …, Written or oral informed consent was obtained from each PLHIV before medical recording.» Is This IRB committee? And Why Written and oral consent? Is this what the IRB committee required?

Authors are using ESOPE database: So are they using secondary data? If so, They should state it.

What is the rationale to classify age as follows: < 30 years, 30-40 years and ≥ 40 years? ( Why not 18-24, 25-35,….). You can notice that authors have to provide the reason. Authors should notice that this way can explain residual confounding.

What is the rational to present Marital status data as polygamous, monogamous and others. We would like to know how never married, divorced and widowed is distributed. And How was the issue per each category.

How many patients were in ESOPE database from January 1, 2006 and December 31, 2012. How many were LTFU during the same period? This is not clear

Authors said : «Of the 8,307 HIV-positive adults contributing to the ESOPE database and starting ART between 2006 and 2012 with CD4 or WHO stage data available at the start of ART, 7,975 individuals returned for their one-month visit and were included in this study (Fig. 2). The individual characteristics including missing» 8,307 were previously LTFU?

I suggest to Authors for key co-variates ( significant in cox model) to present them in Kaplan Meier curve: This will be easy to see the difference. 
Authors remove the Conclusion section but maintained in the abstract?

Conclusion: MAJOR REVISION
