# Supplementary material for: Re-engagement in care of people living with HIV lost to follow-up after initiation of antiretroviral therapy in Mali: Who returns to care?
Source: PLoS One. 2020 Sep 10;15(9):e0238687. doi: 10.1371/journal.pone.0238687 (PMC7482938; doi:10.1371/journal.pone.0238687)
Supplement: Attachment — Submitted filename: Response to Reviewers R2.docx [file pone.0238687.s006.docx]

Review Comments to the Author

Please use the space provided to explain your answers to the questions above. You may also include additional comments for the author, including concerns about dual publication, research ethics, or publication ethics. (Please upload your review as an attachment if it exceeds 20,000 characters)

Reviewer #2: Review report

The Authors did not address adequately to our comments therefore, We are maintaining the decision of major revision. The Authors did not provide adequate justification. This submission is replicating or derive from existing work. The Plos One Policy is clear for this case and Editor has to appreciate.

Authors have published results on LTFU in 2019: and they described LTFU in their papers: The current paper might be concentrated in re-engagement as primary outcome instead of repeating data on LTFU in the same population:

<https://pubmed.ncbi.nlm.nih.gov/30270487-risk-factors-for-loss-to-follow-up-transfer-or-death-among-people-living-with-hiv-on-their-first-antiretroviral-therapy-regimen-in-mali/?from_term=Risk+factors+for+loss+to+follow+up%2C+transfer+or+death+among+people+living+on+their+first+antiretroviral+therapy+regimen+in++Mali&from_pos=1>
The Authors should note that PLOS One publication criteria state that if a submitted study replicates or is very similar to previous work, authors must provide a sound scientific rationale for the submitted work and clearly reference and discuss the existing literature. Submissions that replicate or are derivative of existing work will likely be rejected if authors do not provide adequate justification:

(<https://journals.plos.org/plosone/s/criteria-for-publication#loc-2>)

*Answer: We are also against duplicate publications. However, we do not think that this is the case for the reasons provided below. Factors associated with re-engagement in care are not necessary the same as factors associated with LTFU.*

*We conducted our first study in order to estimate the cumulative incidence of loss of follow-up and to assess risk factors for this event in PLVIH in Mali. We had observed that some of PLHIV lost to follow-up subsequently returned to care with sometimes more than one care interruption during their follow-up and that some did not. Given that Mali, a resource-limited country, does not have an electronic national database, death registries or resources dedicated to active tracing, we wanted to identify among PLHIV with a first interruption of care factors associated with a subsequent return to care.*

*In the resubmitted version, we had already removed from the main text all references to the description of the general population, but this population was described in supplementary material and we referred to the supplementary material in the text. We have now completely deleted this part from the main text in the result section and we removed the supplementary material, and we concentrated in re-engagement in care only.*

*The first two paragraphs of the result section were changed from:*

*"* Of the 8,307 HIV-positive adults contributing to the ESOPE database and starting ART between 2006 and 2012 with CD4 or WHO stage data available at the start of ART, 7,975 individuals returned for their one-month visit and were included in this study (Fig. 2). The individual characteristics including missing data are described in Supplementary Table 1 based on their subsequent follow-up status (‘Remained in care’, ‘LTFU before 12/31/2013’, ‘LTFU after 12/31/2013’, ‘Transfer’ or ‘Death’). The percentages of missing data were 14% for CD4 cell count, 9% for WHO stage, and 15% for educational level. During the study period, 2,302 (29%) PLHIV remained in care, 3,650 (46%) and 1,008 (12%) were lost to follow-up for at least six months during their follow-up before and after 12/31/2013, respectively. Also, during the study period, 595 (8%) and 420 (5%) were known to be transferred to another care centre and to be dead, respectively (S1 Table).

The study included 5,330 (67%) women and median (interquartile range [IQR]) age was 36 (29-43). The most prescribed initial ART regimen contained zidovudine (AZT) or stavudine (d4T) and lamivudine (3TC) plus nevirapine (NVP) or efavirenz (EFV) for 6,106 (77%) individuals. The second most prescribed one contained tenofovir (TDF) and emtricitabine (FTC) or 3TC plus NVP or EFV for 1,332 (17%) individuals. Overall median follow-up was 26 months (IQR, 8-48). Characteristics at the start of ART of the individuals were slightly different according to the region of care and the care centre expertise level (S2 Table).*"*

*To:*

*"* Of the 8,037 HIV-positive adults contributing to the ESOPE database and starting ART between 2006 and 2012 with CD4 or WHO stage data available at the start of ART, 7,975 individuals returned for their one-month visit and 4,658 were lost to follow-up before the database closure (03/31/2015) (Fig. 2). Among those PLHIV lost to follow-up, 3,650 were lost to follow-up before 12/31/2013 and were included in this study (Fig. 2). The study included 2,380 (65%) women and median (interquartile range [IQR]) age was 35 (29-43). The most prescribed initial ART regimen contained zidovudine (AZT) or stavudine (d4T) and lamivudine (3TC) plus nevirapine (NVP) or efavirenz (EFV) for 2,958 (81%) individuals. The second most prescribed one contained tenofovir (TDF) and emtricitabine (FTC) or 3TC plus NVP or EFV for 429 (12%) individuals. Overall median duration of ART before LTFU was 11 months (IQR, 5-22) (S1 Table).*".*

Methods : How did they measure the socio-economic status? We did not found such data in the manuscript. They have reported Professional activity: Did they use that as socio-economic status?

The authors said that, they removed all the socioeconomic terms in their manuscript. Socio-economic terms are important in terms of controlling confounding. They have to provide rationale and how they have define and collect such variables instead of to remove. This is reduce the quality of their analysis.

*Answer*: *As we previously stated, we used the occupation (Professional activity) to measure the socioeconomic status. We do not have income data in the ESOPE database. In the absence of income data, the more conventional indicators of socioeconomic status are completed schooling and occupation (Duncan GJ et al, Am J Public Health 2002; Mackenbach JP et al, N Engl J of Med 2008). Education is the most stable measure of socioeconomic position, because it is normally completed early in adulthood, avoiding most of the problems of reverse causation (Mackenbach JP et al, Lancet Public Health 2019). The educational level collected in ESOPE database is the “The last attained educational level”. However, as suggested we kept the occupation in our final model and changed our results in the paper (see results in the Table 1 below).*

*We changed the sentence in the statistical analyses section, from:*

"The considered individual characteristics at ART initiation were a combined variable of sex and pregnancy, age, WHO stage and CD4 count, period of ART initiation, marital status, last attained educational level and a variable combining the location (Bamako or one of the regions), expertise level (outpatient clinic or hospital) of the care centre and its distance from the individual’s home."

*To*

"The considered individual characteristics at ART initiation were a combined variable of sex and pregnancy, age, WHO stage and CD4 count, period of ART initiation, marital status, last attained educational level, professional activity and a variable combining the location (Bamako or one of the regions), expertise level (outpatient clinic or hospital) of the care centre and its distance from the individual’s home."

*We also added the following sentence in the statistical analyses section:*

*“*Professional activity categories were obtained by combining types and sectors of employment, housewife without any professional activity, public or private sector salaried employment, various non-salaried activities, such as being self-employed, farmer/fisherman, other (unemployed including students) and missing.”

*Of note, we previously mentioned in our discussion that in several studies from sub-Saharan Africa, PLHIV lost to follow-up reported that poverty and the lack of money for transportation cost were barriers to return to care [7, 37-39].*

**Table 1.** Factors associated with re-engagement in care in the 36 months after LTFU among people living with HIV starting ART in Mali subsequent to LTFU (Cox model with imputed data)

| Characteristics | Extended LTFU  N = 1,975 | Return to care  N = 1,675 | Univariable | | Multivariable | |
| --- | --- | --- | --- | --- | --- | --- |
|  | *n (%)* | *n (%)* | HR (95% CI) | *P* | HR (95% CI) | *P* |
| Sex and pregnancy  Men  Non-pregnant women  Pregnant women | 723 (36.6)  1,171 (59.3)  81 (4.1) | 547 (32.7)  926 (55.3)  202 (12.0) | 1  1.04 (0.94-1.15)  2.10 (1.81-2.43) | *< 0.0001* | 1  1.04 (0.91-1.19)  1.56 (1.30-1.86) | *< 0.0001* |
| Age (years)  < 30  [30-40[  ≥ 40 | 551 (27.9)  761 (38.5)  663 (33.6) | 485 (29.0)  673 (40.2)  517 (30.8) | 1  1.02 (0.91-1.14)  0.92 (0.82-1.04) | *0.1980* | 1  1.10 (0.98-1.24)  1.04 (0.91-1.19) | *0.1502* |
| WHO stage and CD4 count (cells/µL) (missing WHO stage, 9%; missing CD4, 14%)  WHO stage 3-4 and CD4 < 200  WHO stage 3-4 and CD4 ≥ 200  WHO stage 1-2 and CD4 < 200  WHO stage 1-2 and CD4 ≥ 200 | 662 (33.5)  274 (13.9)  612 (31.0)  427 (21.6) | 404 (24.1)  208 (12.4)  573 (34.2)  490 (29.3) | 1  1.19 (0.99-1.43)  1.33 (1.17-1.52)  1.54 (1.35-1.75) | *< 0.0001* | 1  1.08 (0.90-1.30)  1.12 (0.98-1.28)  1.23 (1.07-1.41) | *0.0102* |
| Periods of ART initiation  2006-2007  2008-2009  2010-2012 | 598 (30.3)  391 (19.8)  986 (49.9) | 817 (48.8)  479 (28.6)  379 (22.6) | 1  0.96 (0.87-1.07)  0.41 (0.36-0.46) | *< 0.0001* | 1  1.20 (1.06-1.36)  0.56 (0.49-0.64) | *< 0.0001* |
| Marital status  Monogamous^a^  Polygamous  Single  Widowed  Divorced  Missing | 869 (44.0)  358 (18.1)  251 (12.7)  221 (11.2)  97 (4.9)  179 (9.1) | 699 (41.7)  353 (21.1)  226 (13.5)  240 (14.3)  97 (5.8)  60 (3.6) | 1  1.14 (1.01-1.29)  1.09 (0.94-1.25)  1.22 (1.06-1.40)  1.17 (0.95-1.43)  0.50 (0.38-0.64) | *<0.0001* | 1  1.06 (0.94-1.20)  0.99 (0.85-1.15)  1.08 (0.94-1.25)  1.04 (0.85-1.28)  0.57 (0.43-0.77) | *0.0094* |
| Educational level (missing, 15%)  None  Primary/Koranic  Secondary  Higher | 994 (50.3)  578 (29.2)  289 (14.7)  114 (5.8) | 759 (45.3)  525 (31.3)  286 (17.1)  105 (6.3) | 1  1.13 (1.00-1.28)  1.19 (1.03-1.38)  1.13 (0.90-1.42) | *0.0283* | 1  1.03 (0.91-1.17)  1.12 (0.97-1.30)  1.04 (0.81-1.34) | *0.1779* |
| Professional activity^b^  Housewife  Public sector staff  Private sector staff  Self-employed  Farmer/Fisherman  Other  Missing | 1,435 (39.3)  209 (5.7)  691 (18.9)  521 (14.3)  243 (6.7)  433 (11.9)  118 (3.2) | 768 (38.9)  104 (5.3)  341 (17.3)  287 (14.5)  164 (8.3)  223 (11.3)  88 (4.4) | 1  1.09 (0.90-1.33)  1.11 (0.98-1.25)  0.96 (0.84-1.11)  0.63 (0.50-0.79)  1.06 (0.91-1.22)  0.49 (0.34-0.70) | <0.0001 | 1  1.02 (0.82-1.27)  1.04 (0.90-1.20)  1.09 (0.92-1.29)  0.78 (0.61-1.00)  0.98 (0.83-1.16)  1.17 (0.81-1.16) | *0.1477* |
| Regions, care centre & distance (km)  Bamako outpatient clinics  Bamako hospitals & < 5  Bamako hospitals & [5-50[  Bamako hospitals & ≥ 50  Regional outpatient clinics & < 5  Regional outpatient clinics & [5-50[  Regional outpatient clinics & ≥ 50  Regional hospitals & [5-50[  Regional hospitals & ≥ 50 | 834 (42.2)  51 (2.6)  349 (17.7)  52 (2.6)  95 (4.8)  74 (3.8)  37 (1.9)  355 (17.9)  128 (6.5) | 1,005 (60.0)  68 (4.0)  300 (17.9)  55 (3.3)  96 (5.7)  38 (2.3)  21 (1.3)  71 (4.2)  21 (1.3) | 1  1.08 (0.86-1.36)  0.82 (0.73-0.93)  0.97 (0.74-1.26)  0.93 (0.76-1.14)  0.53 (0.39-0.72)  0.58 (0.38-0.88)  0.25 (0.19-0.31)  0.20 (0.13-0.31) | *< 0.0001* | 1  1.22 (0.97-1.54)  0.91 (0.79-1.04)  1.18 (0.92-1.52)  0.96 (0.74-1.23)  0.78 (0.56-1.09)  0.64 (0.41-1.01)  0.36 (0.28-0.47)  0.33 (0.21-0.51) | *< 0.0001* |
| Duration of ART until LTFU (month) and 12-month change in CD4 count (cell/µL)^c^ (missing CD4 at LTFU, 44%)  < 6  [6-12[ and < 50  [6-12[ and ≥ 50  ≥ 12 and < 50  ≥ 12 and ≥ 50 | 711 (36.0)  283 (14.3)  101 (5.1)  641 (32.5)  239 (12.1) | 338 (20.2)  227 (13.6)  270 (16.1)  250 (14.9)  590 (35.2) | 1  1.50 (1.26-1.77)  3.15 (2.66-3.72)  0.90 (0.75-1.08)  3.09 (2.70-3.53) | *< 0.0001* | 1  1.46 (1.23-1.73)  2.61 (2.21-3.08)  0.87 (0.72-1.04)  2.43 (2.12-2.78) | *< 0.0001* |

Abbreviations: LTFU, loss to follow-up; ART, antiretroviral therapy; HR, hazards ratio; CI, confidence interval.  ^a^Married or living with a partner. ^b^Professional activities: professional activity categories combined types and sectors of employment, housewife without any professional activity, public or private sector salaried employment, various non-salaried activities, such as being self-employed, farmer/fisherman, and other (unemployed including students). ^c^Variable combining the duration of ART until LTFU (month) and the estimation of the 12-month change in CD4 count (cells/ µL).

In the method section: Authors should explain why they define periods of ART initiation in such period. Did they do in term of national guidelines?

*Answer: Yes, the periods that we used correspond to the changes of the national guidelines. To clarify, we added the following sentences (underlined) to the study population section:*

“At the end of 2005, first national guidelines on HIV care and ART were released, with subsequent revisions in 2008, 2010 and 2013 according to WHO guidelines. In 2005, guidelines recommended treatment for all PLHIV with CD4 counts ≤ 200 cells/µL, at WHO stage 3 and CD4 counts ≤ 350 cells/µL, or at WHO stage 4. In 2008 and 2010, all PLHIV with CD4 counts ≤ 350 cells/µL or at WHO stage 3-4 had to be treated. Pregnant women beginning ART at WHO stage 1-2 and/or CD4 counts > 350 cells/µL were excluded from this study, because ART initiated for pregnancy was stopped after delivery if the clinical stage and biological results at the time of treatment initiation were not in the range of the indication for treatment in the national guidelines for ART [9].

In Mali, the recommended initial ART regimen for HIV-1-infected individuals consists of two nucleoside reverse transcriptase inhibitors (NRTI), including lamivudine or emtricitabine, plus one non-nucleoside reverse transcriptase inhibitor (NNRTI), nevirapine or efavirenz. In 2010, stavudine was removed from the recommended initial ART regimen. The currently recommended initial ART regimen is tenofovir plus lamivudine and efavirenz [9].”

Authors did not address to this comment: «To analyze the Re-engagement issue: How authors did measure the duration from LTFU and re-engagement: What was the end date since this is the outcome.» Readers will need to know How they measure the duration from LTFU and re-engagement. If the definition is not clear this can introduce bias and misclassification. For the patients who were definitively LTFU during the subsequent three-year follow-up period, How did Authors calculate the duration for them. The start date and End point are not clear: This still some confusion. If this is not clear; Authors should use logistic regression instead of Cox regression?

*Answer: This was explained but perhaps likely not very clearly. To be clearer, we corrected statistical analyses section as follows:*

*The sentence was:*

*"* We assessed factors associated with re-engagement in care in PLHIV lost to follow-up. For this analysis, PLHIV who were lost to follow-up after December 31, 2013 were excluded to allow sufficient time to re-engage in care before the last database update (03/31/2015). The baseline for this analysis was the date of LTFU. Follow-up time was censored at closure of the database or three years after the date of LTFU, whichever occurred first. Factors associated with re-engagement in care were assessed using univariable and multivariable Cox models [13] allowing the comparison of PLHIV who returned to care after LTFU (‘return to care’ group) and those who did not return to care after LTFU (‘extended LTFU’ group). *"*

*We changed it to:*

*“*We estimated the cumulative incidence of re-engagement in care and assessed factors associated with this event in PLHIV lost to follow-up. The baseline for this analysis was the date of LTFU. The time without follow-up was counted from the date of LTFU to the date of re-engagement in care, three years after the date of LTFU or closure of the database (03/31/2015), whichever occurred first. The cumulative incidence of re-engagement in care was estimated using Kaplan-Meier. Factors associated with re-engagement in care were assessed using univariable and multivariable Cox models [13] allowing the comparison of PLHIV who returned to care after LTFU (‘return to care’ group) and those who did not return to care after LTFU (‘extended LTFU’ group).”

Authors said: «We assessed factors associated with re-engagement in care in PLHIV lost to follow-up. For this analysis, PLHIV who were lost to follow-up after December 31, 2013 were excluded to allow sufficient time to re-engage in care before the last database update (03/31/2015). » and «This study included all HIV-1-infected adults, aged ≥ 18 years, who began ART between January 1, 2006 and December 31, 2012 at one of the 16 care centres contributing to the ESOPE database, thus beginning treatment at least two years before the last database update (31 March 2015), and who returned for their one-month visit.» This can make confusion

*Answer: We agree with this comment and we corrected the study population section as follows:*

*From:*

"This study included all HIV-1-infected adults, aged ≥ 18 years, who began ART between January 1, 2006 and December 31, 2012 at one of the 16 care centres contributing to the ESOPE database, thus beginning treatment at least two years before the last database update (31 March 2015), and who returned for their one-month visit."

*To*:

“This study included all PLHIV who were lost to-follow-up before December 31, 2013, to allow sufficient time to re-engage in care before the last database update (03/31/2015). These PLHIV lost to follow-up were selected from all HIV-1-infected adults, aged ≥ 18 years, who began ART between January 1, 2006 and December 31, 2012 at one of the 16 care centres contributing to the ESOPE database, thus beginning treatment at least two years before the last database update (31 March 2015), and who returned for their one-month visit.”

Authors said: «We fitted linear regression, logistic regression, and multinomial regression models for CD4 counts at the start of ART and at LTFU (fourth root), WHO stage (1-2 or 3-4), and educational level (none, primary/koranic, secondary and higher), respectively. » We did not find any results reported to this analysis.

*Answer: This sentence refers to the method used to calculate missing values for PLHIV for whom some data were missing. Then all PLHIV are analyzed and we provided the analyze for all PLHIV.*

Authors said that they did not look for confounding variables. If this is the case, What will be the validity of such study: Did authors assess the internal validity of their study? During this process, authors should analyze type of errors which could occur in their study:

- Random errors (sample size and sampling procedure)

- Selection Bias (LFU in the RCT)

- Information Bias: How the data was measured; (observer, instrument, observed and environment). It can be: error measurement, improper diagnostic criteria, omissions, imprecise measurement. To assess it we use sensitivity and specificity to determine the degree of misclassification (That why we need clear of how they measure the duration from LTFU and re-engagement)

- Confounding: we can control it in two stage:

in design: by randomization, stratification, matching (case-control) and restriction (by inclusion and exclusion criteria)

in Analysis: Stratification or by using multivariate technique

*Answer: We are sorry we were not clear in what we wanted to say. We wanted to say that the study did not focus specifically on identifying the confounders. We assessed in multivariable analyses the association between re-engagement in care and the characteristics of PLHIV lost to follow-up among which potential confounding variables. We thus adjusted on the potential confounders that were available in the database. If we consider social variables, we think that depression and income could be confounding factors for marital status or professional activity, respectively, but we did not have these data. More, we looked for interactions between the variables and took into account the interactions we found in our model. We also checked for multicollinearity. We conducted a multivariable analysis taking into account the relation between potential confounders and re-engagement in care.*

Authors assessed the interactions between several variables without providing the reason of doing that.

*Answer: We now provide the reasons for investigating these interactions. The end of the first paragraph of the statistical section was changed from:*

*"* We assessed the interaction between the duration of ART until LTFU and the estimation of the 12-month change in CD4 counts before LTFU, and the interactions between region of care, care centre expertise level and distance from home to care centre. We also assessed the interactions between the period of ART initiation and the following variables: Sex and pregnancy, age, WHO stage and CD4 count, and distance from home to care centre.*"*

*To:*

“We assessed the interaction between the duration of ART until LTFU and the estimation of the 12-month change in CD4 counts before LTFU because the change in CD4 usually depends on the time on ART. We assessed the interactions between region of care, care centre expertise level and distance from home to care centre because recruitment of PLHIV can be different between the different centres and regions according to distance from home. Given the revisions of national guidelines on HIV care and ART, we also assessed the interactions between the period of ART initiation and the following variables: Sex and pregnancy, age, WHO stage and CD4 count, and distance from home to care centre.”

Confounding factors is what looks like a causal relationship between a supposed factor and an outcome (disease, other event,..) may be due to another factor not taken into consideration.
Manuscripts submitted to PLOS ONE are expected to report statistical methods in sufficient detail for others to replicate the analysis performed. Ensure that results are rigorously reported in accordance with community standards and that the statistical methods employed are appropriate for the study design.

I would request that Authors put publically their dataset for next version.

*Answer: Our dataset can be made available upon request.*

Authors said : «The study was approved by the national AIDS programme in Mali (approval letter available). …, Written or oral informed consent was obtained from each PLHIV before medical recording.» Is This IRB committee? And Why Written and oral consent? Is this what the IRB committee required?

*Answer: The national AIDS program is the competent Institutional Review Board (IRB)for a study on PLHIV. Written and oral consent from any new HIV-positive individual is what the IRB committee requires before the creation of a paper or electronic medical record.*

Authors are using ESOPE database: So are they using secondary data? If so, They should state it.

*Answer: We used data from the ESOPE database. We do not know what are secondary data.*

What is the rationale to classify age as follows: < 30 years, 30-40 years and ≥ 40 years? ( Why not [18-24, 25-35](callto:18-24,%2025-35),….). You can notice that authors have to provide the reason. Authors should notice that this way can explain residual confounding.

*Answer: We added the following sentence in the statistical analyses section:*

“The age at ART initiation was divided into three categories using terciles.”

What is the rational to present Marital status data as polygamous, monogamous and others. We would like to know how never married, divorced and widowed is distributed. And How was the issue per each category.

*Answer: We aimed to have a reasonably small number of categories for the variables. The marital status was divided into three categories (monogamous or living with a partner, polygamous and other (living alone -single, widowed, divorced- and few missing data)).*

*We provided the distribution of other categories (single, widowed, divorced and missing) of marital status in Table 1. Also, in the table below, we show the different categories of marital status (monogamous, polygamous, single, widowed, divorced) were not associated with subsequent re-engagement in care.*

*We added a sentence in the result section (second paragraph of 'factors associated with re-engagement in care’):*

“Conversely, PLHIV who started ART in 2010-2012 (aHR 0.56; 95%CI 0.49-0.64) were less likely to re-engage in care than those initiating ART in 2006-2007 (Table 1). Marital status and professional activity were not associated with re-engagement in care, except farmers/fishermen who were at lower risk of re-engagement in care compared to housewives.

How many patients were in ESOPE database from January 1, 2006 and December 31, 2012. How many were LTFU during the same period? This is not clear

Authors said : «Of the 8,307 HIV-positive adults contributing to the ESOPE database and starting ART between 2006 and 2012 with CD4 or WHO stage data available at the start of ART, 7,975 individuals returned for their one-month visit and were included in this study (Fig. 2). The individual characteristics including missing» 8,307 were previously LTFU?

*Answer: There was an error on the number, it is 8,037 instead of 8,307.*

*We corrected the section.*

I suggest to Authors for key co-variates (significant in cox model) to present them in Kaplan Meier curve: This will be easy to see the difference.

*Answer: We do not think this is a good idea because Kaplan Meier only gives the univariable results of the association between the covariates and return to care, without adjustment on confounders.*

Authors remove the Conclusion section but maintained in the abstract?

*Answer: We did not remove the conclusion in discussion section. In this conclusion we mentioned the factors associated with re-engagement in care and formulated recommendations for the Malian AIDS programme. We just removed the interpretations from the conclusion of the abstract.*

Conclusion: MAJOR REVISION
