# Supplementary material for: Re-engagement in care of people living with HIV lost to follow-up after initiation of antiretroviral therapy in Mali: Who returns to care?
Source: PLoS One. 2020 Sep 10;15(9):e0238687. doi: 10.1371/journal.pone.0238687 (PMC7482938; doi:10.1371/journal.pone.0238687)
Supplement: Attachment — Submitted filename: Response to reviewers R3.docx [file pone.0238687.s007.docx]

Review Comments to the Author

Please use the space provided to explain your answers to the questions above. You may also include additional comments for the author, including concerns about dual publication, research ethics, or publication ethics. (Please upload your review as an attachment if it exceeds 20,000 characters)

Reviewer #3: My only suggestion would be to clarify the definition of LTFU under the "Definitions" paragraph in the methods. This currently states "Loss to follow-up (LTFU) was defined as an interval of at least six months without any clinical appointment for PLHIV not known to be dead or to have been transferred elsewhere". I presume the authors mean "attended visit" -- the term 'visit' is used in the remainder of this paragraph and it would be better to keep it consistent.

*Answer: To remain consistent, we corrected the first paragraph of “Definitions” in the methods section as follows:*

*From:*

"Loss to follow-up (LTFU) was defined as an interval of at least six months without any clinical appointment for PLHIV not known to be dead or to have been transferred elsewhere. The date of LTFU was the date of the first missed scheduled clinical visit [5, 12], as clinician’s first consider an individual to be LTFU at the time of the first missed visit."

*To*:

"Loss to follow-up (LTFU) was defined as an interval of at least six months without any clinical visit for PLHIV not known to be dead or to have been transferred elsewhere. The date of LTFU was the date of the first missed scheduled clinical visit [5, 12], as clinician’s first consider an individual to be LTFU at the time of the first missed visit."
